# The Protective Effect of Magnolol in Osteoarthritis: *In vitro* and *in vivo* Studies

**DOI:** 10.3389/fphar.2019.00393

**Published:** 2019-04-16

**Authors:** Zhi-Chao Hu, Zu-Cheng Luo, Bing-Jie Jiang, Xin Fu, Jiang-Wei Xuan, Xiao-Bin Li, Yu-Jie Bian, Wen-Fei Ni, Ji-Xin Xue

**Affiliations:** ^1^Department of Orthopaedics, The Second Affiliated Hospital and Yuying Children’s Hospital of Wenzhou Medical University, Wenzhou, China; ^2^The Second School of Medicine, Wenzhou Medical University, Wenzhou, China; ^3^Bone Research Institute, The Key Orthopaedic Laboratory of Zhejiang Province, Wenzhou, China

**Keywords:** magnolol, PI3K/Akt/NF-κB, osteoarthritis, interleukin-1 beta, inflammation

## Abstract

Osteoarthritis (OA), defined as a long-term progressive joint disease, is characterized by cartilage impairment and erosion. In recent decades, magnolol, as a type of lignin extracted from *Magnolia officinalis*, has been proved to play a potent anti-inflammatory role in various diseases. The current research sought to examine the latent mechanism of magnolol and its protective role in alleviating the progress of OA *in vivo* as well as *in vitro* experimentations. *In vitro*, the over-production of Nitric oxide (NO), prostaglandin E2 (PGE2), cyclooxygenase-2 (COX-2), inducible nitric oxide synthase (iNOS), tumor necrosis factor alpha (TNF-α), and interleukin-6 (IL-6), induced by interleukin-1 beta (IL-1β), were all inhibited notably by magnolol in a concentration-dependent manner. Moreover, magnolol could also downregulate the expression of metalloproteinase 13 (MMP13) and thrombospondin motifs 5 (ADAMTS5). All these changes ultimately led to the deterioration of the extracellular matrix (ECM) induced by IL-1β. Mechanistically, magnolol suppressed the activation of PI3K/Akt/NF-κB pathway. Furthermore, a powerful binding capacity between magnolol and PI3K was also revealed in our molecular docking research. In addition, magnolol-induced protective effects in OA development were also detected in a mouse model. In summary, this research suggested that magnolol possessed a new therapeutic potential for the development of OA.

## Introduction

As a degenerative joint disease, osteoarthritis (OA) is frequently seen in the elderly population (Appleton, 2018). The pathological hallmark of OA development is the articular cartilage loss, synovitis, subchondral bone recasting, and cartilage hypertrophy (Glyn-Jones et al., 2015; Flemming and Gustas-French, 2017; Scotece et al., 2018). Gender, age, genetics, and other risk factors could also contribute to the musculoskeletal pain and the articular cartilage degeneration (Bennell and Hinman, 2011; Yang et al., 2018). Although the pathophysiology of OA is not fully understood, inflammatory and inflammation-related biomarkers seem to play a critical part (Jotanovic et al., 2012). Meanwhile, it has been demonstrated that the excessive interleukin-1β (IL-1β), as one of the main initiators in the development of OA, exerts harmful effects by up-regulating the production of catabolic factors and pro-inflammatory factors including nitric oxide (NO), matrix metalloproteinases (MMPs), prostaglandin E_2_ (PGE_2_), and thrombospondin motifs (ADAMTS), which ultimately lead to the extracellular matrix (ECM) degradation (Abramson et al., 2001; Verma and Dalal, 2011; Zeng et al., 2015; Zhou et al., 2018). Furthermore, the increased secretion of IL-1β is highly detected in the OA patients’ cartilage, subchondral bone, synovial fluid, and membrane (Moos et al., 1999). Therefore, new effective agents aiming to suppress IL-1β-induced inflammation or IL-1β may provide new regimens to ameliorate the progression of OA.

NF-κB has been described as a master regulator involved in inflammatory response and catabolism (Kumar et al., 2004; Zhang et al., 2018). And it is worth mentioning that NF-κB signaling pathways could be initiated by a broad range of stimuli, such as TNF-α and IL-1β; this eventually stimulates the expression of MMP-1, IL-6, and IL-8 by the phosphorylation of p65 and following translation of p65 into nucleus (Sarkar et al., 2008; Hayden and Ghosh, 2014; Wang W. et al., 2018). Meanwhile, previous studies showed that the PI3K/Akt pathway could be an upstream activator of the NF-κB pathway cascade because it could up-regulate the phosphorylation level of IκBα (Li et al., 2018). Accumulating evidence also demonstrated that the suppression of the PI3K/Akt signaling pathway could lead to the blocking of the activation of NF-kB, which was proved to be therapeutically effective in OA (Reddy et al., 1997; Jin et al., 2018). Thus, we have reasons to believe that the inhibition of PI3K/Akt/NF-κB pathways may be a promising avenue for possible treatment and prevention of OA.

Magnolol, extracted from the Chinese medicinal herb named *Magnolia officinalis*, has a long history for the treatment of several diseases such as diarrhea, anxiety, fever, headache, and asthma (Lee et al., 2011). Nowadays, accumulating evidence indicates that magnolol has a wide range of potent pharmacological activities including antifungal (Jada et al., 2012), anticancer (Li et al., 2015), hepatoprotective, and antioxidant effects (Chen et al., 2009). Previous literatures suggested that magnolol partly mediated inflammatory conditions by repressing the activation of NF-κB pathways in human lung epithelial cells (Chunlian et al., 2014). Rasul et al. (2012) found that magnolol could inhibit the stimulation of the PI3K/Akt in gastric adenocarcinoma cells. Meanwhile, magnolol also markedly down-regulated the secretion of pro-inflammatory cytokines and possessed strong anti-inflammatory effects in mouse macrophages (Lu et al., 2015). Although magnolol’s potential anti-inflammatory role has been extensively investigated, the effect of magnolol remains unclear in OA patient with chondrocytes dysfunction. Therefore, in the present study, we tried to explore the anti-inflammatory role of magnolol in IL-1β-stimulated human chondrocytes and the protective function of magnolol both *in vitro* and *in vivo*. Furthermore, the molecular docking studies were also performed to explore the potential binding mode between magnolol and PI3K protein complex.

## Materials and Methods

### Reagents and Antibodies

Magnolol (purity ≥ 98%) was gained from Herbpurify (Chengdu, China). Cell-Counting Kit-8 (CCK-8) was obtained from Dojindo (Kumano, Japan). Recombinant human IL-1β, collagenase type II, and dimethylsulfoxide (DMSO) were purchased from Sigma Chemical Co. (St. Louis, MO, United States). The goat anti-rabbit and anti-mouse IgG-HRP were purchased from Bioworld (OH, United States), inducible nitric oxide synthase (iNOS) antibody was acquired from Sigma-Aldrich (St Louis, MO, United States), the primary antibody against GADPH and Lamin B1 were acquired from Abcam (CA, United Kingdom). Alexa Fluor^®^488 labeled and Alexa Fluor^®^594 labeled Goat Anti-Rabbit IgG (H+L) second antibody was obtained from Jackson ImmunoResearch (West Grove, PA, United States). Primary antibodies against P-Akt, Akt, PI3K, P-PI3K, COX-2, IκBα, and p65 were obtained from CST (MA, United States). The cell culture reagents were purchased from Gibco (Grand Island, NY, United States). The 4′, 6-diamidino-2-phenylindole (DAPI) was purchased from Beyotime (Shanghai, China).

### Primary Human Osteoarthritis Chondrocytes Culture

Tissue sample collection was following the acknowledgment of the Medical Ethical Committee of the Second Affiliated Hospital and Yuying Children’s Hospital of Wenzhou Medical University (ethic cord: LCKY-2017-29) and the guidelines of the Declaration of Helsinki (World Medical Association [WMA], 2013). The informed consent was acquired from all individuals involved in this research. We obtained samples from 6 patients (three women and three men, aged 60–66 years) who underwent total knee arthroplasty.

We cut hyaline cartilage into pieces and managed it with 2 mg/mL of collagenase II in DMEM/F12 for 4 h at 37°C. Next, we incubated the chondrocytes at a seeding density of 2 × 10^5^ cells/mL in DMEM/F12 supplemented with 1% antibiotic and 10% FBS in an atmosphere of 5% CO_2_ at 37°C. To prevent phenotype loss, we utilized chondrocytes no later than the first passage for consecutive studies.

### Animal Model

Sixty 10-week-old C57BL/6 male wild-type (WT) mice were obtained from the Animal Center of Chinese Academy of Sciences Shanghai, China. As previous work described, the mouse OA models underwent surgical destabilization of the medial meniscus (DMM) (Glasson et al., 2007). Primarily, we anesthetized OA models with intraperitoneal injection of 2% (w/v) pentobarbital (40 mg/kg) and then acquired the joint capsule of right knee medial to the patellar tendon. The medial meniscotibial ligaments were cut with microsurgical scissors. We also performed a surgery containing an arthrotomy with no process of the medial meniscotibial ligament in the left knee joint of models. And the left joint was adopted as a sham group. Ultimately, all of the mice were divided into three groups randomly: magnolol-treated group, vehicle group, and sham group. We acquired approval by the Animal Care and Use Committee of Wenzhou Medical University (ethics code: wydw2014-0129) for experimental procedures. All procedures followed the guidelines for the Care and Use of Laboratory Animals of the National Institutes of Health.

### Experimental Design

*In vitro*, chondrocytes were incubated with 10 ng/mL IL-1β, alone or with magnolol at corresponding concentrations (5, 10, 20 μM) to analyze its anti-inflammatory effects. And we defined an untreated group without the transaction of medial meniscotibial ligament as the control group. Meanwhile, the duration was 2 h to assess the activation of the NF-κB pathway induced by IL-1β, while the duration was extended to 24 h to explore the functional changes.

*In vivo*, mouse models received surgical DMM. Then the magnolol-treated group received magnolol dissolved in saline (20 mg/kg/day) and was administrated by intragastric ways daily for eight continuous weeks (Tian et al., 2018). Meanwhile, models in vehicle group were administered with an equivalent volume of saline. Eventually, all post-surgery mice were sacrificed after 8 weeks, and the articular cartilage samples were collected for histological analysis.

### Cell Viability Assay

The cell counting kit-8 (CCK-8; Dojindo Co., Kumamoto, Japan) was adopted to measure the cytotoxicity of magnolol on chondrocytes. Briefly, we cultured the first-passage chondrocytes in 96-well plates (5 × 10^3^ cell/cm^2^) for 24 h. Then cells were incubated at different concentrations of magnolol (0, 5, 10, 20, 50, 100 μM) for 24 and 48 h. After that, 100 μl of DMEM/F12 solution containing 10 μl of CCK-8 was added to each well of the plate and the chondrocytes were incubated for another 2 h at 37°C. The optical density of the wells was observed at 450 nm wavelength using a spectrophotometer (Thermo Fisher Scientific). All experiments were repeated five times.

### NO, PGE_2_, TNF-α, IL-6 Measurement

We measured the level of NO in the culture medium using Griess reagent (Au et al., 2007). Based on the instructions, commercial ELISA kits (R&D Systems, Minneapolis, MN, United States) were used to measure the concentration of PGE_2_, TNF-α, IL-6, ADAMTS-5, MMP13, Collagen II, and aggrecan in cell culture supernatants. All assays were repeated five times.

### Real-Time PCR

We used TRIzol reagent (Invitrogen) to isolated the total RNA of chondrocytes stimulated with IL-1β (10 ng/ml) and magnolol at different concentrations in 6-cm culture plates. 1 μg of total RNA was reverse transcribed to compound cDNA (MBI Fermentas, Germany). A total 10 μl of reaction volume (5 μl of 2 × SYBR Master Mix, 0.25 μl of each primer and 4.5 μl of diluted cDNA) was adopted for the quantitative real-time PCR (qPCR). Parameters of RT-PCR performed using CFX96 Real-Time PCR System (Bio-Rad Laboratories, CA, United States) were listed as follows: 10 min 95°C, followed by 40 cycles of 15 s 95°C and 1 min 60°C. We collected the cycle threshold (Ct) values and normalized the level of target mRNA to the level of GAPDH. Then we adopted the 2^-ΔΔCt^ method to calculate the level of relative mRNA of each target gene. The primers of COX-2, iNOS, IL-6, and TNF-α were designed using NCBI Primer-Blast Tool^1^; primer’s sequences of the targeted genes were listed as follows: COX-2 (F) 5′-GAGAGATGTATCCTCCCACAGTCA-3′, (R) 5′-GACCAGGCACCAGACCAAAG-3′; iNOS (F) 5′-CCTTACGAGGCGAAGAAGGACAG-3′, (R) 5′-CAGTTTGAGAGAGGAGGCTCCG-3′; IL-6, (F) 5′-GACAGCCACTCACCTCTTCA-3′, (R) 5′-TTCACCAGGCAAGTCTCCTC-3′; TNF-α (F) 5′-GTCAGATCATCTTCTCGA ACC-3′, (R) 5′-CAGATAGATGGGCTCATACC-3′.

### Western Blotting

The protein of chondrocytes was extracted adopting RIPA lysis buffer with 1 mM PMSF (Phenylmethanesulfonyl fluoride). Lysates were put on the ice for 10 min followed by 15 min centrifugation at 12000 rpm 4°C, and then the protein concentration was measured using the BCA protein assay kit (Beyotime). Sodium dodecyl sulfate-polyacrylamide gel electrophoresis (SDS PAGE) and polyvinylidene difluoride (PVDF) membrane were used to separate and transfer 40 ng of protein. After blocked with 5% non-fat milk for 2 h at room temperature, the membranes were incubated with the primary antibody against p65(1:1000), IκB-α(1:1000), iNOS(1:1000), COX-2(1:1000), PI3K(1:1000), P-PI3K(1:1000), AKT(1:1000), P-AKT(1:1000), Lamin-B(1:5000), and GADPH(1:5000) overnight at 4°C. Then subsequent incubation with respective secondary antibodies was performed for 2 h at room temperature. After three times washing using TBST, electrochemiluminescence plus reagent (Invitrogen) was used to visualize the blots. Finally, we adopted Image Lab 3.0 software (Bio-Rad) to quantify the intensity of these blots.

### Immunofluorescence

For collagen II staining, we seeded cells in glass plates in six-well plates and then treated them with single 10 ng/ml IL-1β or 10 ng/ml IL-1β combined with 20 μM magnolol for 24 h after incubated with serum-starved medium overnights. For p65 staining, we reduced the duration of IL-1β and magnolol treatment to 2 h. After treatments, PBS was used to rinse the samples three-times before fixation using 4% paraformaldehyde. Then the samples were permeabilized using the 0.1% Triton X-100 diluted in PBS for 15 min. Later, cells were blocked with 5% bovine serum albumin for 1 h at 37°C, rinsed with PBS and incubated with primary antibodies against collagen α (1:200) and p65 (1:200) overnight at 4°C. After washing the glass plates on the next day, chondrocytes were incubated with subsequent second antibodies (1:400) for 1 h at room temperature and marked with DAPI for 5 min. Finally, slides were viewed under the fluorescence microscope (Olympus Inc., Tokyo, Japan), and the fluorescence intensity was tested using Image J software 2.1 (Bethesda, MD, United States).

### Molecular Modeling

The PI3K (PDB ID: 3LJ3) was adopted for molecular docking design (Khanfar et al., 2015). Protein acquired from RCSB PDB was prepared for the subsequent docking^2^. Then the lowest energy conformations were adopted for molecular docking via default parameters using PyMoL (version 1.7.6). We also used the AutoDockTools (version 1.5.6) to analyze the protein-ligand docking study, which could provide the binding affinity of the ligand into the binding pocket residues. All of the 3-D images were performed in UCSF PyMoL, while the 2-D interactions were generated in Ligplus v.1.4.

### Histopathologic Analysis

We used Safranin-O/Fast Green to stain the slides of each joint. Then, we took advantage of a microscope to photograph the morphologic changes of chondrocytes and surrounding tissue; we also adopted the Osteoarthritis Research Society International (OARSI) scoring system for medial femoral condyle and medial tibial plateau as described previously (Glasson et al., 2010) to evaluate the destruction of articular cartilage. Fifteen mice each group were adopted.

### Statistical Analysis

The analyses were repeated at least five times. *Mean* ± standard deviation (*SD*) was adopted to present the results. SPSS 20.0 was used to perform statistical analyses. For comparison between control and treatment groups, data were calculated using one-way analysis of variance (ANOVA) followed by the Tukey’s test. Non-parametric data were analyzed using the Kruskal–Wallis *H*-test. *P*-values less than 0.05 were considered statistically significant (Tang et al., 2017; Wang H. et al., 2018; Zheng et al., 2019).

## Results

### Effect of Magnolol on Human Chondrocytes Viability

**Figure 1:**
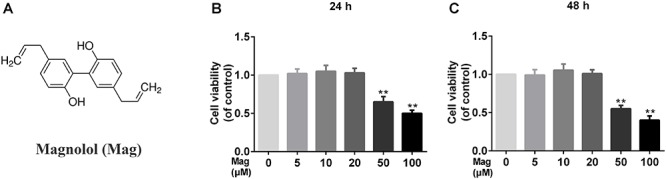
Effects of magnolol on the cell viability of chondrocytes. **(A)** Chemical structure of magnolol. **(B,C)** The cytotoxic effect of magnolol on chondrocytes was determined at various concentrations for 24 and 48 h using a CCK8 assay. The values presented are the means ±*SD*. Of five independent experiments. ^∗^*P* < 0.05, ^∗∗^*P* < 0.01 vs. control group, *n* = 5.

Figure 1A showed the chemical structure of magnolol. We used the CCK-8 assay to assess the viability of human chondrocytes treated with magnolol. Incubated with magnolol (0, 5, 10, 20, 50, and 100 μM), the cellular viability of the cells was measured using CCK-8 assay for 24 and 48 h. Next, we calculated cell cytotoxicity as the percentage of the control group. CCK8 results illustrated that magnolol within 20 μM was not cytotoxic to human chondrocytes after 24 or 48 h (Figure 1B,C). Thus, we adopted 5, 10, 20 μM magnolol to conduct the subsequent experiments.

### Magnolol Suppressed the IL-1β Induced Expression of COX-2, iNOS, PGE_2_, NO, TNF-α, and IL-6 in Human Chondrocytes

**Figure 2:**
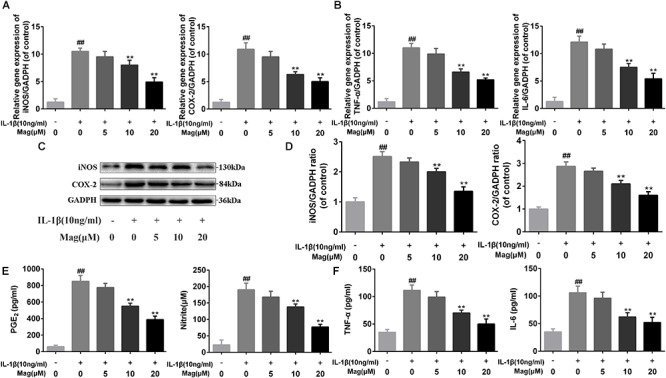
Magnolol inhibits inflammatory effect in chondrocyte. The mRNA expression of iNOS, COX-2, TNF-α, and IL-6 were measured by real-time PCR **(A,B)**. The protein expressions of iNOS and COX-2 in chondrocytes treated as above were visualized by western blot **(C)**, quantified in **(D)**. Effect of magnolol on IL-1β-induced PGE_2_, NO, TNF-α, and IL-6 production in human OA chondrocytes **(E,F)**. The data in the figures represent the averages ± *SD*. Significant differences among different groups are indicated as ^##^*P* < 0.01, vs. control group; ^∗^*P* < 0.05, ^∗∗^*P* < 0.01, vs. IL-1β alone treatment group, *n* = 5.

Next RT-PCR, ELISA, and Western blot analysis were performed to study the effect of magnolol in the production of COX-2, iNOS, PGE2, NO, TNF-α, and IL-6. Figure 2A,C,D demonstrated that magnolol, in a manner with 5, 10, and 20 μM, restrained the up-regulation of IL-1β (10 ng/ml)-induced expression of COX-2 and iNOS, but we didn’t find any statistical significance between IL-1β alone treatment group and the 5 μM magnolol treatment group at both mRNA and protein levels. Moreover, under the IL-1β stimulation, the secretion of endogenous PGE_2_ and NO was up-regulated. In Figure 2E, the treatment with magnolol led to the decrease of PGE_2_ expression and NO generation in a dose-dependent manner, while there was no significant difference at the concentration of 5 μM magnolol treatment. What’s more, as shown in Figure 2B,F, TNF-α and IL-6 production were inhibited in a concentration-dependent inhibition, which was detected by qRT-PCR analysis and ELISA; however, no statistical significance was found between the group treated with a concentration of 5 μM magnolol and the group treated with IL-1β alone. Altogether, these data illustrated that the secretion of these mediators of inflammation could be inhibited by magnolol treatment at mRNA and protein levels in a dose-dependent manner, especially at the concentration of 10 and 20 μM (*P* < 0.05).

### Magnolol Ameliorated ECM Deterioration in Human OA Chondrocytes

**Figure 3:**
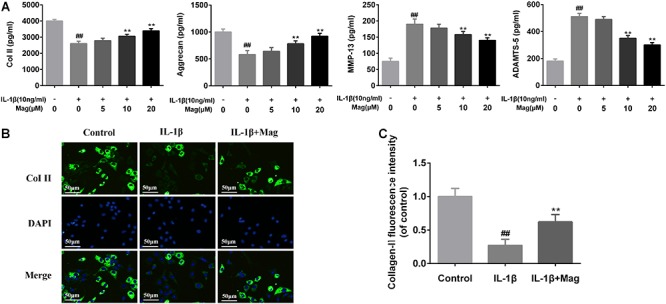
Magnolol inhibits ECM degradation in human OA chondrocytes. The protein expression of Col II, aggrecan, MMP-13, and ADAMTS-5 in culture medium from chondrocytes treated as described above in **(A)**. Typical collagen-II **(B)** was detected by immunofluorescence combined with DAPI staining for nuclei (original magnification × 100, scale bar: 50 μm). The fluorescence intensity of collagen-II **(C)** was determined using Image J software. Values represent the averages ±*SD*. Significant differences among different groups are indicated as ^##^*P* < 0.01, vs. control group; ^∗^*P* < 0.05, ^∗∗^*P* < 0.01, vs. IL-1β alone treatment group, *n* = 5.

We next analyzed magnolol’s influence on aggrecan and collagen-II degradation induced by IL-1β in human OA cells. Figure 3A showed that magnolol activated aggrecan and collagen-II, but inhibited ADAMTS-5 and MMP-13 expression in a dose-dependent manner, especially at 10 and 20 μM with *P* < 0.05. Meanwhile, as described in Figure 3B,C, the outcomes of immunofluorescence staining of collagen-II-active protein appeared to be consistent with the ELISA results. Taken together, all these results above indicated that the treatment with magnolol ameliorated ECM deterioration in human OA chondrocytes.

### Magnolol Exerted a Protective Effect on IκBα Degradation and p65 Translocation in Human OA Chondrocytes

**Figure 4:**
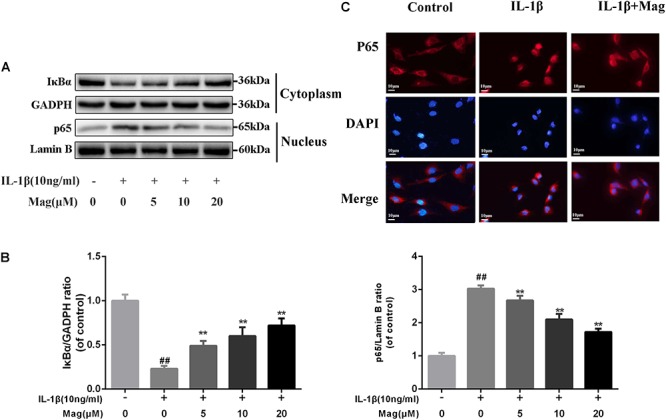
Effect of magnolol on IL-1β-induced NF-κB activation. The protein expressions of IκBα in cytoplasm and p65 in nuclear in chondrocytes treated as above were visualized by western blot **(A)**, and quantified in **(B)**. **(C)** The nuclei translocation of p65 was detected by the immunofluorescence combined with DAPI staining for nuclei (original magnification × 400, scale bar: 10 μm). The data in the figures represent the averages ±*SD*. Significant differences among different groups are indicated as ^##^*P* < 0.01, vs. control group; ^∗^*P* < 0.05, ^∗∗^*P* < 0.01, vs. IL-1β alone treatment group, *n* = 5.

NF-κB signaling pathway plays a core part involving in the production of inflammatory mediators. Thus, we performed western blot analysis to scrutinize the protein levels of IκBα in the cytoplasm and p65 expression in the nucleus in OA cells to further comprehend the anti-inflammatory effects of magnolol. IL-1β markedly promoted IκBα decay and induced the translocation of p65 into the nucleus. Nevertheless, as Figure 4A,B described, these effects were outstandingly suppressed by the pretreatment with magnolol in a concentration-dependent manner. We also conducted immunofluorescence staining of p65 to detect the translocation of p65 from the cytoplasm to nucleus. In the control group, the p65-active proteins were chiefly localized in chondrocytes’ cytoplasm. Yet under the stimulation of IL-1β, the p65-active proteins underwent noticeable translocation from the cytoplasm to the nucleus. Obviously, magnolol extremely ameliorated the translocation of p65 (Figure 4C). Collectively, the results indicated that magnolol could exert a protective effect in human OA chondrocytes by inhibiting the NF-κB pathway activation.

### Magnolol Inhibited the PI3K/Akt Signal Pathway Activation in Human OA Chondrocytes

**Figure 5:**
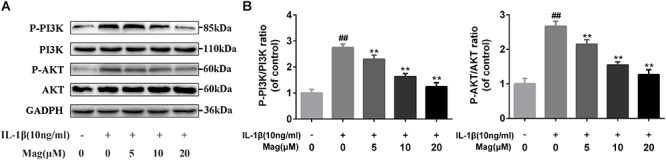
Effect of magnolol on IL-1β-induced PI3K/Akt activation in human OA chondrocytes. The protein expression levels of P-PI3K, PI3K, P-Akt, and Akt were determined by western blot and quantification analysis **(A,B)**. Data are expressed as mean ± *SD*. Significant differences among different groups are indicated as ^##^*P* < 0.01, vs. control group; ^∗^*P* < 0.05, ^∗∗^*P* < 0.01, vs. IL-1β alone treatment group, *n* = 5.

PI3K plays a crucial role in the Akt activation and is reported to involve in the IL-1β-induced inflammatory response. Therefore, to comprehend the effects of magnolol on the PI3K/Akt axis, we adopted western blot to examine the phosphorylation of PI3K and Akt. Magnolol pretreatment was found to inhibit the phosphorylation of PI3K and Akt in a concentration-dependent manner (5, 10, and 20 μM) (Figure 5A,B). Besides, statistical significance was found in all these results (*P* < 0.01). However, the highest level of inhibitory effect was reached at the concentrations of 20 μM. All in all, these results indicated that magnolol down-regulated the expression of the phosphorylation of PI3K and Akt in a concentration-dependent manner.

### Molecular Docking

**Figure 6:**
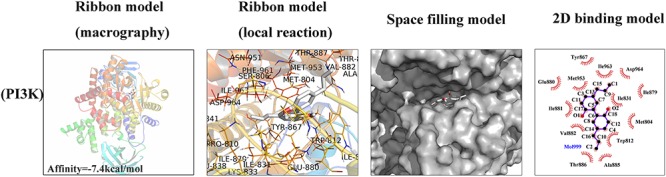
Magnolol was docked with PI3K structure. Docking studies were performed as described in the Section “Materials and Methods.” The protein residues are shown in a ribbon model and 2D binding model. The proposed binding pose of magnolol shows CH- π interactions with PI3K structure. Space filling model shows the binding of magnolol in the inhibitory binding pocket.

To investigate whether magnolol has a direct affinity with related upstream protein in PI3K/Akt/NF-κB cascades, we conducted a molecular docking study to explore the potential binding mode between magnolol and PI3K protein complex (Khanfar et al., 2015). The chemical structure of magnolol, 4-Allyl-2-(5-allyl-2-hydroxy-phenyl)phenol, is described in Figure 1A. In Figure 6, the macro structure and the local interaction were revealed in a ribbon model. In addition, a space-filling model was analyzed to illuminate the 3D structure of magnolol in the related active pocket. Based on the molecular docking with PI3K structure, detailed analysis showed that the phenyl group of magnolol formed the CH-π interactions with the residue TYR-867 and TRP-812 of PI3K, respectively, with a high affinity of -7.4 kcal/mol. Strong hydrophobic interaction force was shown in the 2D binding model; around the ligands molecules were Ala885, Glu880, Met953, Tyr867, Asp964, Ile879, Ile881, Ile831, Ile963, Met804, Val882, Thr886, and Trp812, which together formed the hydrophobic interaction. All the procedures were analyzed with the PyMOL and Ligplus.

### Magnolol Ameliorated OA Development in the DMM Mouse Model

**Figure 7:**
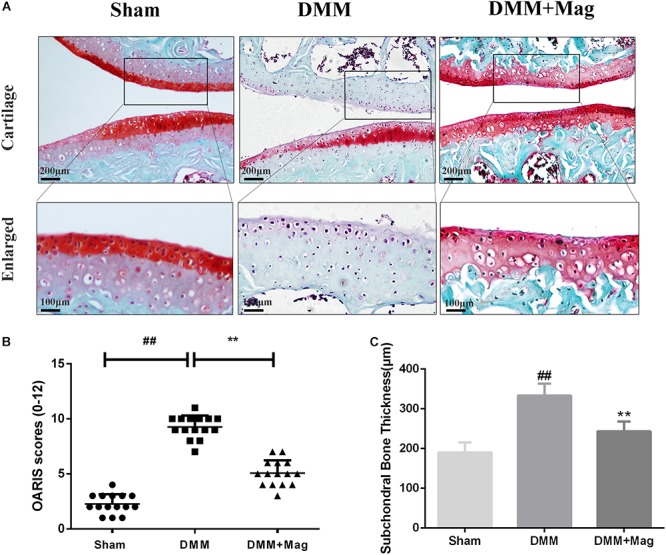
Magnolol ameliorates OA development in mouse DMM model *in vivo*. **(A)** Representative S-O staining of cartilage from different experimental groups at 8 weeks post-surgery (original magnification × 100 or × 400, scale bar: 200 or 100 μm). **(B)** Diagrams showed the OARIS scores of cartilage. **(C)** Diagrams showed the subchondral bone plate thickness. The data in the figures represent the averages ±*SD*. Significant differences among different groups are indicated as ^##^*P* < 0.01, vs. sham group; ^∗∗^*P* < 0.01, vs. DMM group, *n* = 15.

*In vivo*, to study the protective effects of magnolol in the development of OA, surgically induced mouse models of DMM were set up. The models were intragastric administrated 20 mg/kg magnolol dissolved in saline or vehicle (saline alone) for 8 weeks once daily. And Safranin-O/Fast Green staining was used to perform histological analysis of OA for cartilage. Osteoarthritis Research Society International (OARSI) scores were adopted for the quantitative analysis. As shown in Figure 7A, in the sham control group, samples appeared positive red staining with smooth cartilage surface. Compared with the sham control group, the OA group illustrated noticeable hypocellularity, cartilage cauterization, and massive proteoglycan degradation. Compared with the OA group, the magnolol group presented a significantly smoother surface of cartilage. Meanwhile, the *mean* and *SD* of OARSI scores in each group were also calculated, respectively. As shown in Figure 7B, the OARSI scores of the OA group were higher than that at sham control group (Sham 2.267 ± 0.884 vs. DMM 9.267 ± 1.033, *P* < 0.01), which was consistent with the Safranin-O/Fast Green staining. Conversely, the magnolol group showed lower OARSI scores than the OA group (DMM 9.267 ± 1.033 vs. DMM + Magnolol 5.067 ± 1.163, *P* < 0.01). In addition, magnolol was found the association with the reduction of the surgery-induced subchondral bone thickness (Sham 189.7 ± 25.1 vs. DMM 332.9 ± 29.8 vs. DMM + Magnolol 243.2 ± 24.6, all *P* < 0.01, Figure 7C). Altogether, the results above suggested that *in vivo* magnolol alleviated OA development via restraining the devastation of cartilage surface.

## Discussion

Osteoarthritis is known as a widespread aging-related joint disorder in elder adults (Heijink et al., 2012). Currently, most OA treatment strategies only temporarily target pain relief and neglect to extenuate OA deterioration (Zeng et al., 2018). Meanwhile, the use of multiple non-surgical regimens was restricted for many aspects such as limited efficacy, recurrent side effects, and variable rates of success. Thus, a milder agent with a certain molecular target is in urgent need to palliate cartilage degradation. Magnolol, isolated from *M. officinalis*, has been demonstrated related to a broad spectrum of anti-inflammatory effects (Chunlian et al., 2014). However, its exact mechanisms and positive roles in OA chondrocytes dysfunction remain unclear. In this work, we detected that magnolol exerted a chondroprotective effect by targeting the PI3K/Akt/NF-κB signaling pathway. Magnolol played a significant role in the NF-κB pathway by the regulation of PI3K/Akt. Similarity, *in vivo* study, it was shown that OA development was ameliorated by magnolol in mice.

Previous studies confirmed that NF-κB pathways played a vital part in OA’s pathogenesis by regulating inflammatory cytokines and mediator (Kumar et al., 2004). Normally, IκBα is phosphorylated and degraded once activated by IL-1β, subsequently resulting in the translocation of p65. In the nucleus, p65 participates in the secretion of cytokines, catabolic enzymes, and inflammatory mediators. Among all the factors above, iNOS catalyzes NO, inducing the secretion of MMPs and inhibiting collagen II and proteoglycan synthesis to promote ECM degradation (Arasapam et al., 2006). PGE_2_ is an inflammatory mediator produced from IL-1β-induced endogenous arachidonic acid by COX-2 and also stimulates the expression of MMPs and ADAMTS5 (Hardy et al., 2002). MMP-13, as a subgroup of collagenases member, is one of the most significant MMPs associated with the catabolism of cartilage (Takeda, 2016). What’s more, ADAMTS5 is considered to play a crucial role in aggrecan’s cleavage in the pathogenesis of the OA (Takeda, 2016). Therefore, a mediator targeting ADAMTS5 and MMP-13 seems to be a valuable regimen in OA development. In this work, magnolol was found to suppress the excessive production of NO, PGE_2_, IL-6, TNF-α as well as the up-regulation of iNOS and COX-2 at both the mRNA and protein level. Moreover, we investigated that magnolol inhibited the secretion of MMP-13 and ADAMTS5 and the loss of aggrecan and type II collagen in human OA cells. All these data suggested that magnolol suppressed IL-1β-induced inflammatory response via blockage of NF-κB pathway in OA.

**Figure 8:**
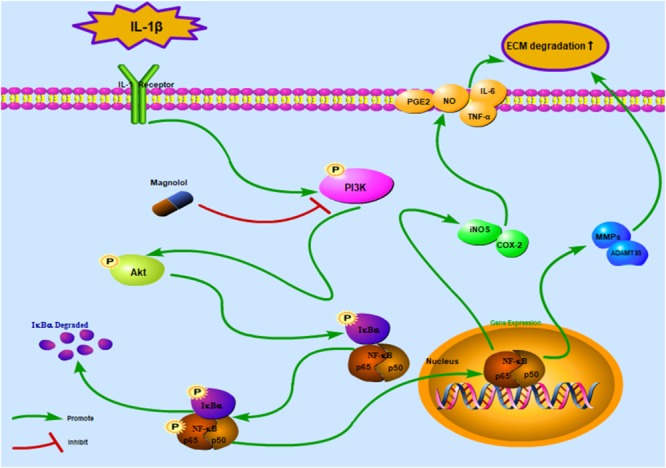
Schematic illustration of the potential protective effects of magnolol in osteoarthritis development. Red arrows indicate the inhibiting effects. Green arrows indicate the promoting effects.

Previous studies demonstrated that the PI3K/Akt pathway was associated with ECM and cellular alterations in the pathogenesis of OA (Chen et al., 2013). Upon stimuli by receptors such as cytokine receptors, the membrane protein PI3K could directly or indirectly induce the phosphorylation of Akt; and phosphorylated Akt activates the key subunit of NF-κB (p65) to phosphorylated p65 and then increases MMPs and COX-2 production by chondrocytes via the activation of its downstream pathway – NF-κB (Shakibaei et al., 2007; Busch et al., 2012). In addition, repression of the PI3K/Akt signaling pathway has been conceived as a regimen for the OA treatment. However, some studies showed contrast results of the PI3K/Akt/NF-κB pathway, which may be partly due to the different experimental conditions and cellular environment (Zhang et al., 2007). What’s more, some researchers believed that the PI3K/Akt signaling pathway may play an important role as a negative feedback regulator that limits pro-inflammatory responses (Patruno et al., 2012). That’s to say, the activation of the PI3K/Akt may be associated with the suppression of the NF-κB pathway. Thus, further studies are still needed to provide actual scientific evidence. In this research, magnolol was found to greatly inhibit the activation of PI3K/Akt induced by IL-1β in human OA cells. The result was supported by the former work which suggesting that magnolol exerted inhibitory effects in human prostate cancer cells via inhibiting PI3K/Akt pathway (Lee et al., 2009). Altogether, the former researches, as well as our findings indicated that magnolol alleviated IL-1β-induced inflammation through inhibiting the activation of PI3K/Akt/NF-κB in OA cells. Meanwhile, the underlying mechanism was shown specifically in Figure 8. The molecular docking studies were performed to explore the potential binding mode between magnolol and PI3K protein complex. The optimized structure of magnolol was docked into the active site of PI3K with ligand WYE (PDB Code: 3LJ3). The crystallographic ligand was extracted from the active site, and the residues within a certain radius around the PI3K molecule were defined as the active pocket. Actually, magnolol occupied the active pocket of PI3K and would competitively inhibit the activation of PI3K (Tsou et al., 2010). Molecular docking analysis showed that magnolol directly occupied the active pocket of PI3K by CH-π interactions with a rather high affinity. The result showed that magnolol suppressed the process of PI3K’s phosphorylation. Although potential upstream or bypass mechanism might exist, our results indicated that in a way magnolol could attenuate the activation of PI3K/Akt/NF-κB pathway by directly inhibiting the activation of PI3K.

The DMM mouse model has been adopted previously to explore similarities between human and animal model OA (Glasson et al., 2007). In DMM mouse models, the mice experienced the loss of chondrocytes, cartilage destruction, and calcification. However, the usage of magnolol ameliorated these detrimental courses, indicating that magnolol could palliate the progress of OA.

## Conclusion

This work demonstrated that magnolol significantly inhibited IL-1β-induced PI3K/Akt/NF-κB pathway activation to attenuate inflammation and catabolism in human OA chondrocytes. Meanwhile, molecular docking results showed that magnolol occupied the active pocket of PI3K, which was consistent with the results found *in vitro*. Furthermore, in DMM-induced OA model, protective effects of magnolol on chondrocytes were also observed. Taken together, the results above may support the use of magnolol as a potential therapeutic regimen for OA.

## Data Availability

The raw data supporting the conclusions of this manuscript will be made available by the authors, without undue reservation, to any qualified researcher.

## Author Contributions

W-FN and J-XX conceived and designed the experiments. Z-CH and Z-CL performed the experiments. Z-CH and B-JJ performed the statistical analysis and wrote the manuscript. J-XX, X-BL, XF, and Y-JB provided assistance with experiments. All authors discussed the results and approved the final manuscript.

## Conflict of Interest Statement

The authors declare that the research was conducted in the absence of any commercial or financial relationships that could be construed as a potential conflict of interest.

## Footnotes

1 https://www.ncbi.nlm.nih.gov/tools/primer-blast/

2 https://www.rcsb.org/
